# Genome-wide scan of the effect of common nsSNPs on colorectal cancer survival outcome

**DOI:** 10.1038/s41416-018-0117-7

**Published:** 2018-08-21

**Authors:** Evropi Theodoratou, Susan M Farrington, Maria Timofeeva, Farhat VN Din, Victoria Svinti, Albert Tenesa, Tao Liu, Annika Lindblom, Steven Gallinger, Harry Campbell, Malcolm G Dunlop

**Affiliations:** 1 Centre of Global Health Research, Usher Institute of Population Health Sciences and Informatics, University of Edinburgh, Teviot Place, Edinburgh, EH8 9AG UK; 2 Colon Cancer Genetics Group and Academic Coloproctology, Institute of Genetics and Molecular Medicine, University of Edinburgh and MRC Human Genetics Unit Western General Hospital Edinburgh, Edinburgh, EH4 2XU UK; 3 The Roslin Institute and Royal (Dick) School of Veterinary Studies, University of Edinburgh, Easter Bush, EH25 9RG UK; 4 Department of Molecular Medicine and Surgery, Karolinska Institute and Department of Clinical Genetics, Karolinska University Hospital, Solna, SE-17176 Stockholm, Sweden; 5 Lunenfeld-Tanenbaum Research Institute, Mount Sinai Hospital, Toronto, ON Canada

**Keywords:** Cancer epidemiology, Cancer genetics

## Abstract

**Background:**

We conducted a genome-wide scan to identify non-synonymous SNPs (nsSNPs) that might influence survival after a diagnosis of colorectal cancer (CRC).

**Methods:**

We genotyped 7679 nsSNPs in 1939 Scottish patients from the Scottish Colorectal Cancer Study recruited soon after a CRC diagnosis and prospectively followed for survival outcomes. All-cause and CRC-specific survival analyses were conducted using Cox proportional hazard models adjusted for stage, age and sex for all cancer cases, after cancer type stratification and assuming additive and recessive models of inheritance. For all the SNPs that had a *p*-value < 0.10 a meta-analysis was performed combining the results of the discovery set and a replication set of 899 Scottish CRC patients. The *p*-value threshold of significance was set as at *p* < 10^−8^.

**Results:**

897 and 894 nsSNPs were associated with all-cause and CRC-specific mortality, respectively, at a *p*-value level < 0.10 in the discovery set. Meta-analysis of the results from the discovery and replication sets was performed overall and for cancers of colon and rectum separately and none of the variants reached a *p*-value < 10^−8^.

**Conclusions:**

This large scale well-powered analysis demonstrates that common nsSNPs are not associated with CRC prognosis overall.

## Introduction

Despite improvements in survival rates from colorectal cancer (CRC) over the last 25 years, deaths from CRC still account for ~10% of all cancer deaths in the UK (Cancer Research UK, 2014). Globally, CRC is a significant cause of cancer mortality with 694,000 deaths per annum (8.5% of total).^1^ Tumour stage at diagnosis currently provides the most prognostic value, described by loco-regional and distant spread.^2^ Alongside age and gender effects, lifestyle factors such as physical activity and body mass index influence particular characteristics of the tumour systemic inflammatory response and impact on cancer survival^3,4^ However, there is also evidence of familial concordance for survival in a number of cancers, including CRC, suggesting that heritable genetic variation may contribute to prognosis.^5^ A number of studies have generated associations between survival outcome and polymorphic genetic variants^6^ and in others with combinations of genotype and particular treatments^7,8^ In a previous study we examined whether Single nucleotide polymorphism (SNP)s influencing CRC risk had any effect on survival after diagnosis, and found that none of ten common genetic variants identified by genome-wide association studies (GWAS) were associated with survival from CRC,^9^ (8q24 (rs6983267),^10,11^ 8q23.3 (rs16892766, *EIF3H*),^12^ 10p14 (rs10795668),^12^ 11q23 (rs3802842),^13^ 15q13 (rs4779584),^14^ 18q21 (rs4939827, *SMAD7*),^13,15^ 14q22.2 (rs4444235, *BMP4*),^16^ 16q22.1 (rs9929218, *CDH1*),^16^ 19q13.1 (rs10411210, *RHPN2*),^16^ and 20p12.3 (rs961253)^16^), although more recent studies have had conflicting and inconsistent results—minor allele of rs4939827 (SMAD7) associated with reduced overall and CRC-specific survival;^17^ five SNPs (rs961253, rs355527, rs4464148, rs6983267 and rs10505477) associated with survival of stage III disease;^18^ rs4444235 significantly associated with survival in CRC patients;^19^ patients homozygous for the minor allele (AA genotype) of rs9929218 had a poorer overall survival rate;^20^ studies from East Asians found rs4779584 association with reduced risk of CRC mortality;^21^ rs1321311 (CDKN1A) and rs10411210 (RHPN2) associated with surgically resected CRC,^22^ and most recently, GG genotype of rs6983267 and the CC genotype of rs1957636 were significantly associated with poorer survival outcomes.^23^ Whilst other studies have utilised GWAS approaches to identify biomarker of therapeutic response.^24^

SNPs that alter the encoded amino-acid sequence (non-synonymous SNPs or nsSNPs), and SNPs mapping within the 5′ and 3′ untranslated regions of genes (that have a direct impact on gene expression) are more likely to have a functional impact, but they are less prevalent in the population.^25^ Furthermore a large proportion of the SNPs identified as statistically significantly associated with CRC risk by GWAS (at GWAS *p*-value threshold), map to regions of the genome outside gene boundaries.^25^ A previous study found no association between CRC risk and nsSNPs identified by a gene-centric genome-wide scan.^26^ Two more studies searched for high-impact mutations within the exome, a highly enriched subset of the genome in which disease-causing mutations are most likely to reside and both studies concluded that recurrent, low-frequency coding variants account for only a minority of the unexplained heritability of CRC.^27,28^

In this study, we interrogated, for the first time, the hypothesis that nsSNPs may affect CRC prognosis itself. We genotyped a large prospectively collected population-based cohort of CRC patients in Scotland (discovery and replication cohorts) for all common nsSNPs to determine any effect on all-cause or CRC-specific mortality.

## Methods

### Description of study patient population—Scottish cohorts

All CRC patients in the Scottish Colorectal Cancer Study (SOCCS; discovery cohort SOCCS 1, replication cohort SOCCS 2) were of Scottish ancestry (defined as parents and all grandparents residing in Scotland).^29^ The work received ethical and management approvals from the MultiCentre Research Ethics committee for Scotland, 18 Local Research Ethics committees, 18 Caldicott guardians and 16 NHS Trust management committees, and all participants provided written informed consent.

Patients were recruited between 1999 and 2006. Recruitment took place as soon as possible after a confirmed diagnosis of adenocarcinoma of large bowel epithelium (typically within 3–7 months of diagnosis, median = 150 days, IQR = 89–243 days), in order to minimise survival bias. We recruited 52% of all CRC cases arising in Scotland during the recruitment period and 98.4% of the recruited cases were finally included in the SOCCS study (3417 CRC cases). Details of case recruitment have been described in more detail elsewhere.^9,11,15^ In brief, research staff were based in the main surgical centres throughout Scotland and ascertainment occurred as soon after admission as possible and clinically appropriate. The main exclusions were as follows: patient death before ascertainment, patient too ill to participate, case was a recurrence of CRC or patient unable to give informed consent due to learning difficulties or other medical conditions. Patients with a personal or family history consistent with a dominant polyposis syndrome or Lynch Syndrome (HNPCC) were excluded from this analysis. Sequence analysis was undertaken to detect carriers of DNA mismatch repair gene mutations and the two common *MUTYH* mutations were assessed for bi-allelic events.^30,31^ All such carriers were also excluded from analysis. In all, genotype data were available for 3017 CRC cases. Cases were excluded from the survival analysis for the following reasons: insufficient DNA for DNA amplification (43 cases), genotyping failure (1 case), previously unrecognised carriers of another susceptibility allele (5 cases), gender discrepancies between phenotype database and genotype (10 cases), date of diagnosis prior to study initiation date (6 cases), missing data (AJCC stage: 98 cases; date of diagnosis: 14 cases), duplicates (2 cases). Therefore, in all 2838 cases were included in the survival analysis. Of these cases, 1939 CRC cases constituted the discovery set (SOCCS 1) and the remaining 899 CRC cases constituted the replication set SOCCS 2. This discovery/replication separation we applied followed the same Phase I/Phase II separation we used in our previous GWAS in CRC risk analysis.^13^

### Genotyping, quality control, variant selection

Three aliquots of 10 ml of blood were collected from each case in one acid citrate dextrose and two sodium ethylenediamin tetra-acetic acid (EDTA) tubes. DNA was extracted from one of the EDTA tubes using Nucleon BACC2 kit (GE Healthcare). Median DNA yield on samples was 327 µg (range: 50–1197 µg). Quality control procedures included spectrophotometric readings of every sample (either A260/280 or PicoGreen™), agarose gel electrophoresis of uncut and restriction enzyme cut DNA from 2% of samples and a control PCR on 1% of samples.^13^

Genotyping for nsSNPs was performed using HumanNS-12 Genotyping BeadChip in 1939 CRC patients.^32,33^ SNPs were selected for this panel by screening public databases for all annotated nsSNPs and it included 11460 nsSNPs.^33^ After we excluded SNPs that failed genotyping (*n* = 3325) and SNPs with a minor allele frequency (MAF) of <1% (*n* = 456), 7679 nsSNPs were genotyped in the discovery set. Of these 7679 nsSNPs, 3888 were genotyped in the replication set SOCCS 2 (Illumina Infinium HumanHap300 and HumanHap240S arrays encompassing ~550,000 genome-wide SNPs), a further 3254 were imputed to 1000 Genome Project (phase 1 integrated release 3, March 2012) with imputation quality info > 0.3 and 537 were not available. Throughout genotyping was performed using the same quality control filters as described by us previously.^13^

### Survival analysis

The CRC cases that were included both in the discovery and replication sets were observed until death or 30th June 2011 (censored date), whichever came first. For 2771 CRC cases (97.6%) date of diagnosis (incidence date) was provided by the Scottish Cancer Registry (SCR). All incidence dates were cross-checked with date of first pathology record and date of definitive clinical diagnosis, which was taken as start of treatment (operation, radiotherapy or decision for palliative therapy). In 67 cases (2.4%), the date of first pathology report collected at recruitment was used as the cancer incidence date. Death certificates were provided by the General Register Office (GRO) for Scotland. There were 810 deaths from the start of recruitment to the censored date in the discovery set and 345 deaths in the replication cohort SOCCS 2. The cause of death was determined by a physician examining all death certificates and there were 610 deaths that were due to CRC (75% of all deaths) in the discovery set and 317 CRC deaths in the replication set (92% of all deaths). The protocol devised for deciding whether the death was related to CRC is presented in Supplementary Box 1. In all cases, assignment of cause of death was blinded to the genotype of the deceased subject. Participant records were linked to SCR and to GRO through the Community Health Index (CHI), which is a NHS population-based register of all individuals who are registered with a general practitioner in Scotland (95% completeness).^34^

To determine the cancer stage according to the American Joint Committee on Cancer (AJCC) system the following procedure was followed. For patients from the South East Scotland (SCAN) region, computerised tomography scans were requested and individually checked for evidence of metastasis. For patients from the West and North of Scotland (WoSCAN and NoSCAN) regions, respectively, the consultants of individual patients were contacted by letter requesting staging information and clarification of metastasis status for their patients. For the remaining cases individual general practitioners were contacted by letter.

All statistical analyses were undertaken using the R software (version 3.3.3) and the statistical package Intercooled STATA version 10.0 (Stata Corp, College Station, Texas). *T*-test Pearson *χ*^2^ test were used to test differences in mean age, sex and AJCC stage between deceased and survived/ censored cases. A Cox proportional hazards model adjusted for AJCC stage, age and sex was used to calculate hazards ratios (HRs) between the SNPs and death. All-cause and CRC-specific survival analyses were also run after site stratification (colon versus rectum) and for different genetic models (additive and recessive). The proportional hazards assumption was checked by fitting a model that included time-dependent covariates, which were created by interactions between the predictors (the SNP, age, sex and AJCC) and survival time. For all the SNPs that had a *p*-value < 0.10 in the discovery set, a meta-analysis was performed combining the results of the discovery and replication set. We planned to type any SNPs that had a meta-analysis *p*-value of <10^−8^ in three additional cohorts (Scottish replication cohort 2, Canadian replication cohort and Swedish replication cohort) and to perform a second meta-analysis. However no SNP passed this threshold. To assess the credibility of the genetic associations, we considered the Bayesian False Discovery Probability (BFDP)^35^ and the False Positive Report Probability (FPRP).^36^ Both the BFDP and FPRP are used to assess the noteworthiness of an observed association. For variants that were found to be statistically significantly associated with risk of CRC in any of the genetic models (at *p* < 10^−4^), the BFDP was estimated using the R package “gap”. The BFDP and FPRP thresholds were set up to be equal to 0.20. We calculated BFDP and FPRP values for three levels of prior probabilities: at a medium (0.10), low (10^–3^) and at a very low prior level (10^–4^). For FRPR all calculations were done assuming statistical power to detect an odds ratio of 1.5. Finally, power calculation was done using the R statistical package “SurvSNP” implementing the approach as described by Owzar et al. 2012.^37^

## Results

Supplementary Tables 1 and 2 present summary statistics by mortality group (all-cause and CRC-specific) of factors influencing survival separately for the discovery and replication sets. AJCC stage was strongly associated with all-cause and CRC-specific mortality in the discovery and replication sets. Numbers of persons at risk, numbers of events and follow-up information at selected time points for the discovery and replication cohorts are presented in Supplementary Table 3. The clinical characteristics of the cases from SCAN, WoSCAN and NoSCAN for the Scottish cohorts are presented in Supplementary Table 4. MAF distribution of the 7679 SNPs is shown in Supplementary Figure 1 (13.4% of variants exhibited a MAF ≤ 0.05; MAF was 0.05–0.20 for 35.7% of variants and MAF > 0.20 for 50.9% of nsSNPs). We evaluated the inflation of test statistics for the nsSNP. As expected we observed significant inflation for both models (recessive and additive). Despite of the significant inflation, we believe that it is very unlikely it is caused by population stratification or other bias. The SOCCS study was previously used in multiple GWAS on CRC risk^13,38^ and we have never observed any evidence of population stratification or inflation in the analysis. However, we do believe that the observed inflation is caused by results enriched for true associations, since the array contains only non-synonymous variants, which could drive the association.

In Table 1 we present the results of the meta-analysis of the discovery and replication cohorts for those variants that had a *p*-value < 10^−4^. In Supplementary Table 5, we present the effect estimates separately in the discovery and replication cohort for each variant presented in Table 1.

**Table 1:** SNPs associated with all-cause and CRC mortality in additive and recessive models at a *p*-value level < 10^−4^ in all cancers and in colon and rectal cancer separately

Model	Site	Name	CHR	Position	Minor allele	MAF	HR (95% CI)	*p*-value	*I* ^2^	FPRP 0.1	FPRP 0.001	FPRP 0.00001	BFDP 0.1	BFDP 0.001	BFDP 0.00001	Variant type	Protein allele	Gene
All cause mortality																		
REC	COLON	rs1805016^a^	16	27363606	G	0.06	10.43 (3.29, 33.06)	6.7E-05	0	0.55	0.99	1.00	0.80	1.00	1.00	Missense variant	S/A	IL4R
REC	COLON	rs637186	11	61125134	A	0.09	5.35 (2.37, 12.07)	5.4E-05	0	0.31	0.98	1.00	0.67	1.00	1.00	Missense variant	H/R	CD5
CRC-specific mortality																		
ADD	RECTUM	rs9320001^a^	18	62147091	G	0.23	1.43 (1.20, 1.71)	7.8E-05	0	0.001	0.10	0.92	0.03	0.78	1.00	Missense variant	H/D	PIGN
REC	ALL	rs12574508^a^	11	9832230	C	0.12	2.57 (1.66, 3.97)	2.1E-05	0	0.02	0.73	1.00	0.16	0.95	1.00	Missense variant	Q/E	SBF2
REC	RECTUM	rs7258236	19	6760963	C	0.22	2.49 (1.62, 3.83)	3.1E-05	0	0.03	0.75	1.00	0.17	0.96	1.00	Missense variant	N/D	SH2D3A

^a^Imputed with Rsq > 0.98 in replication set

The Cox proportional hazards regression analysis of the discovery set (adjusted for AJCC stage, age and sex) returned 897 nsSNP associations with all-cause mortality under additive model of inheritance at a *p*-value level of less than 0.10. Of these, 846 nsSNPs were either genotyped or imputed in the replication set SOCCS 2. Meta-analysis of the discovery and replication sets was performed. None of the SNPs were statistically significant at *p*-value < 10^−4^. Similarly, none of the nsSNPs were associated with all-cause mortality at a *p*-value < 10^−4^ in the analysis restricted to cases with colon and cases with rectal cancers only.

Similarly, 894 nsSNPs were associated with CRC-specific mortality under additive model of inheritance at a *p*-value level of less than 0.10. Of these, 847 were either genotyped or imputed in the replication set. Meta-analysis of the discovery and replication sets was performed. None of the SNPs were statistically significant at *p*-value < 10^−4^ when all cancer cases were combined. However, rs9320001 on chromosome 18 was associated with CRC-specific mortality (*p* < 10^−4^) in patients with rectal cancer (Table 1).

We further performed analysis assuming a recessive model of inheritance. 731 nsSNP were associated with all-cause mortality at a *p*-value level of less than 0.10. Of these 692 nsSNPs associated with all-cause mortality at a *p* ≤ 0.10 were either genotyped or imputed in the replication set SOCCS 2. None of the variants reached assigned significance level of *p*-value < 10^−4^ in the meta-analysis of the discovery and replication sets for all cancer cases. However, rs637186 on chromosome 11 and rs1805016 on chromosome 16 were associated with all-cause mortality (*p* < 10^−4^) in patients with colon cancer (Table 1).

753 nsSNPs were associated with CRC-specific mortality under recessive model of inheritance at a *p*-value level of less than 0.10. Of these, 706 nsSNPs associated with CRC-specific mortality at a *p*-value ≤ 0.10 were either genotyped or imputed in the replication set. Meta-analysis of the discovery and replication sets was performed and the results of the nsSNPs with a *p*-value < 10^−4^ are presented in Table 1. The variant rs12574508 in chromosome 11 was associated with CRC-mortality at *p*-value < 10^−4^ in the meta-analysis of the discovery and replication sets for all cancer cases. In addition, rs7258236 on chromosome 19 was associated with CRC mortality (*p* < 10^−4^) in patients with rectal cancer (Table 1).

Supplementary Figure 2 show the relationship between effect sizes (measured by HR, taking the reciprocal for HRs < 1.0) and MAF for the 7679 SNPs. We tested for association between stage at presentation and genotype for each of the SNPs that were associated with all-cause or CRC-specific mortality at a *p*-value level of <10^-4^ (Supplementary Table 5). Genotypes at all SNPs showed no relationship with stage at representation. Finally, we fitted a model including a time-dependent form of the covariates (AJCC, sex and age) to check the proportional hazards assumption. The assumption was true for the SNP, age and sex covariates, but not for the AJCC stage covariate. We therefore re-ran the analysis stratified by stage after age and sex adjustment. Results for SNPs that were associated with all-cause and/or CRC-specific mortality after AJCC stratification at a *p*-value level of <10^-4^ are presented in Supplementary Tables 6 and 7.

To minimise survival bias due to the time gap between day of diagnosis and recruitment in the study (median time to recruitment = 150.0), we run all analyses after adjusting for left truncation by using time to recruitment to create survival objects in R (Supplementary Table 8**)**. None of the SNPs were statistically significant at *p*-value < 10^−8^.

This study has reasonable power (≥80%) to detect HR of 2.0 for individual SNPs with MAF > 5% in the analysis including all cancer cases and stratified by colon and rectum (Supplementary Figure 3). For individual variants exerting lesser effect size (HR > 1.5) the study had ≥80% power for variants (MAF > 10%) in the analysis that includes all colon and rectal cancers together (Supplementary Figure 4). We accept that these power estimates are at the upper range of the estimates, but given that the genetic architecture of survival outcome for CRC is unknown, any more complex analysis of power would be highly speculative. Hence, these seem plausible estimates and emphasise that much larger sample sizes would be required, given the complexity of measured, and unmeasured, variables that impact on cancer survival outcomes.

## Discussion

### Main findings

GWAS of tagging SNPs have so far identified around 40 loci that contribute to the heritable component of CRC risk.^39^ These genetic variants are common in populations of European ancestry and their identification has provided new insights into the aetiology of CRC.^40^ An alternative and simple approach is to employ a genome-wide strategy based on coding variants, as this can highlight immediately the genes involved in altering disease risk and progression. In the current study we examined the associations between a set of 7679 common (MAF ≥ 1%) nsSNPs and all-cause or CRC-specific mortality in a discovery set of 1939 CRC cases. We performed a meta-analysis of the discovery and a replication set of 899 CRC cases (replication set SOCCS 2) for all the SNPs with a *p*-value < 0.10. None of the genotyped nsSNPs had a *p*-value < 10^−8^.

### Strengths and limitations

The strengths of this study include the systematic and prospective nature of the collected dataset of CRC cases from almost all hospitals in Scotland, and the relatively large sample size of CRC cases compared to other published CRC survival studies. Cases were recruited as soon as possible after diagnosis in order to limit survival bias amongst those recruited and maximise the person years of follow up. We therefore consider that this systematic study of cancer experience from across a whole country provides results that are broadly representative of the general population. In addition, data relevant to the survival analysis were of high quality, since they were obtained from the comprehensive Scottish registries GRO and SCR (which have been shown to have high levels of data quality, validity and completeness) after linkage of our participants with their databases using CHI numbers. In addition, considerable effort was expended to determine and validate the coded AJCC stage for every case through review of individual pathology, clinical and imaging records. Finally any positive associations were explored in four replication cohorts.

A limitation of our study includes the possibility of a relative under-representation of extremely ill patients at presentation, who died very soon afterwards, even within the same hospital admission. It is therefore possible, though unlikely, that external validity of results may be limited for CRC patients of advanced AJCC stage or those who present with a complication, such as bowel obstruction or perforation. Additional co-morbidity might also have influenced the survival of non-recruited subjects, independent of cancer stage at presentation. It is unlikely that treatment differences between hospitals could have affected survival because CRC clinical management is standardised in Scotland through the Scottish Intercollegiate Guidelines Network (SIGN; http://www.sign.ac.uk/). The objective of SIGN is to improve the quality of health care for patients in Scotland by reducing variation in practice and outcome. Finally, the HumanNS-12 Genotyping BeadChip array covers only about 30% of the common non-synonymous variants presented in the UK population as compared to the exome array and could offer complete whole exome coverage of all possible functional variants and indels. Therefore, population-specific custom arrays as well as exome and genome sequencing may be a way forward to identify recurrent rare genetic variation of moderate to large effect sizes.

## Conclusion

In conclusion, although germline determinants of survival outcome have enormous potential to shed new light on the processes of cancer metastasis and the mechanisms leading to cancer death, we did not identify any nsSNPs that were convincingly linked to all-cause or CRC mortality. Although, our study had sufficient power to detect moderate and strong effects (HR > 2.0) of common nsSNPs, it had limited power to detect very rare variants or variants of modest effect size. Therefore there is an urging need in collaborative efforts to increase sample size and power of genetic association studies on CRC survival.

## Electronic supplementary material

Supplementary materials

## Availability of data and material

Summary data supporting the results reported in this article can be requested by emailing the corresponding author.
