# Supplementary material for: Genome-wide scan of the effect of common nsSNPs on colorectal cancer survival outcome
Source: Br J Cancer. 2018 Aug 21;119(8):988–93. doi: 10.1038/s41416-018-0117-7 (PMC6203849; doi:10.1038/s41416-018-0117-7)
Supplement: Supplementary file 1 — Supplementary materials [file 41416_2018_117_MOESM1_ESM.docx]

**Supplementary material**

**Supplementary Box 1** Protocol to establish cause of death

The following criteria were set to ensure that all death certificates were examined in a standardised manner. If the death certificate stated the presence of ‘metastasis’ or ‘carcinomatosis’, then the cause of death was presumed to be due to colorectal cancer. If two primary cancers were noted on the death certificate, then the death was attributed to colorectal cancer unless the certificate clearly stated that the cause of death was due to the a non-colorectal cancer. When the death certificate stated that the cancer had been inoperable tumour, then the cause of death was noted as related to colorectal cancer. The cause of death was attributed to colorectal cancer if the certificate stated that the cause of death was due to a visceral or intra-abdominal complication which may have been directly related to colorectal cancer or the treatment of the disease. All death certificates were checked independently by one clinical and one non-clinical investigator to ensure that there was agreement as to the cause of death.

**Supplementary table 1** Summary statistics of all-cause mortality for factors influencing survival

| ***All-cause mortality*** | ***All cases*** | ***Deceased***  ***Cases*** | ***Survived/***  ***Censored cases*** | ***p-value*** |
| --- | --- | --- | --- | --- |
| **Discovery set** | **N=1939** | **N=810** | **N=1129** |  |
| *Age (years)* | 66.3 (8.90) | 67.39 (8.97) | 65.55 (10.82) | <5x10^-5^ |
| *Sex* |  |  |  |  |
| Men | 1175 | 527 | 648 |  |
| Women | 764 | 283 | 481 | 0.001 |
| *AJCC stage* |  |  |  |  |
| 1 | 369 | 81 | 288 |  |
| 2 | 704 | 216 | 488 |  |
| 3 | 603 | 276 | 327 |  |
| 4 | 263 | 237 | 26 | <5x10^-4^ |
| *Site* |  |  |  |  |
| Colon | 1118 | 464 | 654 |  |
| Rectum | 572 | 234 | 338 | 0.8146 |
|  |  |  |  |  |
| **Replication set**  **SOOCS 1** | **N=899** | **N=345** | **N=554** |  |
| *Age (years)* | 49.60 (5.94) | 49.26 (6.51) | 49.81 (5.56) | 0.18 |
| *Sex* |  |  |  |  |
| Men | 455 | 169 | 286 |  |
| Women | 444 | 176 | 268 | 0.44 |
| *AJCC stage* |  |  |  |  |
| 1 | 172 | 20 | 152 |  |
| 2 | 273 | 57 | 216 |  |
| 3 | 288 | 111 | 177 |  |
| 4 | 166 | 157 | 9 | <5x10^-4^ |
| *Site* |  |  |  |  |
| Colon | 475 | 178 | 297 |  |
| Rectum | 356 | 141 | 215 | 0.5315 |

**Supplementary table 2** Summary statistics for CRC-specific mortality for factors influencing survival

| ***CRC- mortality*** | ***All cases*** | ***Deceased***  ***Cases*** | ***Survived/***  ***Censored cases*** | ***p-value*** |
| --- | --- | --- | --- | --- |
| **Discovery set** | **n=1939** | **n=610** | **n=1329** |  |
| *Age (years)* | 66.32 (8.90) | 65.98 (9.33) | 66.48 (8.69) | 0.26 |
| *Sex* |  |  |  |  |
| Men | 1175 | 389 | 786 |  |
| Women | 764 | 221 | 543 | 0.05 |
| *AJCC stage* |  |  |  |  |
| 1 | 369 | 31 | 338 |  |
| 2 | 704 | 127 | 577 |  |
| 3 | 603 | 223 | 380 |  |
| 4 | 263 | 229 | 34 | <5x10^-4^ |
| *Site* |  |  |  |  |
| Colon | 1118 | 337 | 781 |  |
| Rectum | 572 | 187 | 385 | 0.2837 |
| **Replication set**  **SOCCS 1** | **N=899** | **N=317** | **N=582** |  |
| *Age (years)* | 49.60 (6.63) | 49.09 (6.63) | 49.87 (5.52) | 0.06 |
| *Sex* |  |  |  |  |
| Men | 455 | 155 | 300 |  |
| Women | 444 | 162 | 282 | 0.45 |
| *AJCC stage* |  |  |  |  |
| 1 | 172 | 11 | 161 |  |
| 2 | 273 | 47 | 226 |  |
| 3 | 288 | 103 | 185 |  |
| 4 | 166 | 156 | 10 | <5x10^-4^ |
| *Site* |  |  |  |  |
| Colon | 475 | 168 | 307 |  |
| Rectum | 356 | 127 | 229 | 0.9274 |

**Supplementary table 3** Kaplan-Meier life table analysis for the discovery and replication sets

| **Time**  **(years)** | **No at**  **risk** | **Deaths** | **Cumulative**  **survival rate** | **Cumulative**  **death rate** |
| --- | --- | --- | --- | --- |
| **Discovery set** |  |  |  |  |
| 0-1 | 1939 | 72 | 0.963 | 0.037 |
| 1-2 | 1867 | 192 | 0.864 | 0.136 |
| 2-3 | 1675 | 158 | 0.782 | 0.218 |
| 3-4 | 1517 | 107 | 0.727 | 0.273 |
| 4-5 | 1410 | 80 | 0.686 | 0.314 |
| 5-6 | 1330 | 65 | 0.652 | 0.348 |
| 6-7 | 1238 | 75 | 0.611 | 0.389 |
| 7-8 | 1091 | 29 | 0.593 | 0.407 |
| 8-9 | 820 | 27 | 0.568 | 0.432 |
| 9-10 | 419 | 4 | 0.559 | 0.441 |
| 10-11 | 69 | 1 | 0.548 | 0.452 |
| 11-12 | 32 | 0 | 0.548 | 0.452 |
| 12-13 | 13 | 0 | 0.548 | 0.452 |
| **Replication**  **set SOCCS 1** |  |  |  |  |
| 0-1 | 899 | 47 | 0.948 | 0.052 |
| 1-2 | 852 | 89 | 0.849 | 0.151 |
| 2-3 | 763 | 77 | 0.763 | 0.237 |
| 3-4 | 686 | 47 | 0.711 | 0.289 |
| 4-5 | 639 | 30 | 0.677 | 0.323 |
| 5-6 | 609 | 24 | 0.651 | 0.349 |
| 6-7 | 584 | 14 | 0.635 | 0.365 |
| 7-8 | 535 | 9 | 0.623 | 0.377 |
| 8-9 | 418 | 4 | 0.616 | 0.384 |
| 9-10 | 290 | 1 | 0.613 | 0.387 |
| 10-11 | 170 | 2 | 0.605 | 0.395 |
| 11-12 | 117 | 1 | 0.597 | 0.403 |
| 12-13 | 37 | 0 | 0.597 | 0.403 |

**Supplementary Table 4** Clinical characteristics of the cases from SCAN, WoSCAN and NoSCAN for the Scottish cohorts

| **Clinical characteristics** | **SE Scotland**  **(SCAN)** | **W Scotland**  **(WoSCAN)** | **N Scotland**  **(NoSCAN)** | **p-value^^[[1]](#footnote-1)^^** |
| --- | --- | --- | --- | --- |
| **Discovery set** | **N=550** | **N=820** | **N=569** |  |
| *All-cause mortality* |  |  |  |  |
| Deceased | 217 (39.45%) | 369 (45.00%) | 224 (39.37%) |  |
| Survived/ censored | 333 (60.55%) | 451 (55.00%) | 345 (60.63%) | 0.05 |
| *CRC mortality* |  |  |  |  |
| Deceased | 171 (31.09%) | 273 (33.29%) | 166 (29.17%) |  |
| Survived/ censored | 379 (68.91%) | 547 (66.71%) | 403 (70.83%) | 0.26 |
| *Age (years)* | 66.17 (8.88) | 66.51 (9.30) | 66.20 (8.30) | 0.73 |
| *Sex* |  |  |  |  |
| Men | 332 (60.36%) | 510 (62.20%) | 333 (58.52%) |  |
| Women | 218 (39.64%) | 310 (37.80%) | 236 (41.48%) | 0.38 |
| *AJCC stage* |  |  |  |  |
| 1 | 99 (18.00%) | 148 (18.05%) | 122 (21.44%) |  |
| 2 | 217 (39.45%) | 286 (34.88%) | 201 (35.33%) |  |
| 3 | 154 (28.00%) | 263 (32.07%) | 186 (32.69%) |  |
| 4 | 80 (14.55%) | 123 (15.00%) | 60 (10.54%) | 0.05 |
| *Follow up time (years)* | 6.56 (3.01) | 6.17 (3.03) | 6.64 (2.98) | 0.007 |
| **Replication**  **Set SOCCS 1** | **N=245** | **N=404** | **N=250** |  |
| *All-cause mortality* |  |  |  |  |
| Deceased | 102 (41.63%) | 155 (38.37%) | 88 (35.20%) |  |
| Survived/ censored | 143 (58.37%) | 249 (61.63%) | 162 (64.80%) | 0.34 |
| *CRC mortality* |  |  |  |  |
| Deceased | 93 (37.96%) | 141 (34.90%) | 83 (33.20%) |  |
| Survived/ censored | 152 (62.04%) | 263 (65.10%) | 167 (66.80%) | 0.53 |
| *Age (years)* | 49.37 (5.73) | 49.54 (5.98) | 49.90 (6.10) | 0.59 |
| *Sex* |  |  |  |  |
| Men | 110 (44.90%) | 211 (52.23%) | 134 (53.60%) |  |
| Women | 135 (55.10%) | 193 (47.77%) | 116 (46.40%) | 0.11 |
| *AJCC stage* |  |  |  |  |
| 1 | 37 (15.10%) | 84 (20.79%) | 51 (20.40%) |  |
| 2 | 68 (27.76%) | 129(31.93%) | 76 (30.40%) |  |
| 3 | 88 (35.92%) | 123(30.45%) | 77 (30.80%) |  |
| 4 | 52 (21.22%) | 68 (16.83%) | 46 (18.40%) | 0.33 |
| *Follow up time (years)* | 6.47 (3.59) | 6.90 (3.58) | 7.14 (3.50) | 0.10 |

Supplementary Table 5 AJCC distribution according to SNP genotype in the discovery set (SNPs that were found to be associated with all-cause mortality or CRC mortality at a p-value <10^-4^)

| **SNP** | **Genotype** | **AJCC** | | | |
| --- | --- | --- | --- | --- | --- |
|  |  | **1** | **2** | **3** | **4** |
| **rs1805016** | TT | 338 | 624 | 546 | 229 |
|  | TG | 30 | 78 | 54 | 32 |
|  | GG | 1 | 2 | 3 | 2 |
|  | *p-value* | *0.4441* |  |  |  |
| **rs637186** | GG | 305 | 584 | 490 | 217 |
|  | GA | 58 | 117 | 108 | 43 |
|  | AA | 6 | 3 | 5 | 3 |
|  | *p-value* | *0.5364* |  |  |  |
| ***rs9320001*** | CC | 219 | 410 | 342 | 159 |
|  | GC | 134 | 252 | 220 | 89 |
|  | CG | 16 | 42 | 41 | 15 |
|  | *p-value* | *0.7699* |  |  |  |
| **rs12574508** | GG | 291 | 560 | 465 | 204 |
|  | GC | 72 | 132 | 128 | 56 |
|  | CC | 6 | 12 | 10 | 3 |
|  | *p-value* | *0.9255* |  |  |  |
| **rs7258236** | TT | 223 | 440 | 376 | 176 |
|  | TC | 132 | 226 | 204 | 75 |
|  | CC | 14 | 38 | 23 | 12 |
|  | *p-value* | *0.4087* |  |  |  |

**Supplementary Table 6** SNPs associated with all-cause mortality at a p-value level <10^-4^ by AJCC stage

| **SNP name** | **Model** | **Site** | **AJCC 1** |  | **AJCC 2** |  | **AJCC 3** |  | **AJCC 4** |  |
| --- | --- | --- | --- | --- | --- | --- | --- | --- | --- | --- |
|  |  |  | **HR**  **(95% CI)** | **p-value** | **HR**  **(95% CI)** | **p-value** | **HR**  **(95% CI)** | **p-value** | **HR**  **(95% CI)** | **p-value** |
| **rs1805016** | REC | Colon | 7.01 (0.88, 55.49) | 0.0652 | NA | NA | 11.85 (1.53, 91.47) | 0.02 | 15.23 (1.86, 124.61) | 0.01 |
| **rs637186** | REC | Colon | 2.76 (0.63, 11.99) | 0.1761 | 0 (0, Inf) | 0.9978 | 8.74 (2.08, 36.69) | 0.0031 | 2.13 (0.52, 8.76) | 0.2934 |

**Supplementary Table 7** SNPs associated with CRC- mortality at a p-value level <10^-4^, by AJCC stage

| **SNP name** | **Model** | **Site** | **AJCC 1** |  | **AJCC 2** |  | **AJCC 3** |  | **AJCC 4** |  |
| --- | --- | --- | --- | --- | --- | --- | --- | --- | --- | --- |
|  |  |  | HR  (95% CI) | p-value | HR  (95% CI) | p-value | HR  (95% CI) | p-value | HR  (95% CI) | p-value |
| rs9320001 | ADD | Rectum | 1.11 (0.58, 2.11) | 0.7581 | 1.34 (0.85,2.13) | 0.2113 | 1.59 (1.20,2.12) | 0.0014 | 1.29 (0.96,1.73) | 0.0969 |
| rs12574508 | REC | Colorectal Cancer | 4.72 (1.44, 15.52) | 0.0105 | 1.63 (0.61, 4.42) | 0.3324 | 2.89  (1.48, 5.62 | 0.0018 | 3.1549 (1.07, 9.28) | 0.0368 |
| rs7258236 | REC | Rectum | 1.98 (0.24, 16.2) | 0.5224 | 2.02 (0.86, 4.74) | 0.1074 | 2.57 (1.12, 5.94) | 0.0267 | 4.4 (0.81, 23.79) | 0.0857 |

**Supplementary Table 8 SNPs associated with all-cause and CRC mortality in additive and recessive models at a p-value level <10^-4^ in all cancers and in colon and rectal cancer separately after taking left truncation into account.**

| Model | Site | name | CHR | Position | Minor allele | MAF | HR | P value | I2 | FPRP 0.1 | FPRP 0.001 | FPRP 0.00001 | BFDP 0.1 | BFDP 0.001 | BFDP 0.00001 | Protein allele | Gene |
| --- | --- | --- | --- | --- | --- | --- | --- | --- | --- | --- | --- | --- | --- | --- | --- | --- | --- |
|  |  |  |  |  |  |  | (95% CI) |  |  |  |  |  |  |  |  |  |  |
| All-cause mortality | | | | | | | | | | | | | | | | | |
| REC | RECTUM | rs7258236 | 19 | 6760963 | C | 0.2238 | 2.32(1.54-3.5) | 5.50E-05 | 0 | 0.03 | 0.75 | 1.00 | 0.18 | 0.96 | 1.00 | N/D | SH2D3A |
| CRC-specific mortality | | | | | | | | | | | | | | | | | |
| ADD | ALL | rs4526148* | 5 | 1081652 | C | 0.4979 | 0.42(0.27-0.65) | 8.75E-05 | 0.19 | 1.00 | 1.00 | 1.00 | 0.26 | 0.98 | 1.00 | A/T | SLC12A7 |
| REC | ALL | rs12574508* | 11 | 9832230 | C | 0.1163 | 2.51(1.62-3.87) | 3.51E-05 | 0.00 | 0.03 | 0.77 | 1.00 | 0.19 | 0.96 | 1.00 | Q/E | SBF2 |
| REC | RECTUM | rs7258236 | 19 | 6760963 | C | 0.2238 | 2.5(1.62-3.83) | 3.00E-05 | 0.00 | 0.03 | 0.75 | 1.00 | 0.17 | 0.96 | 1.00 | N/D | SH2D3A |

*Imputed with Rsq > 0.90 in replication set

Supplementary Figure 1 Distribution of minor allele frequency for the SNPs analyses

| **Plot A** | **Plot B** |
| --- | --- |
| 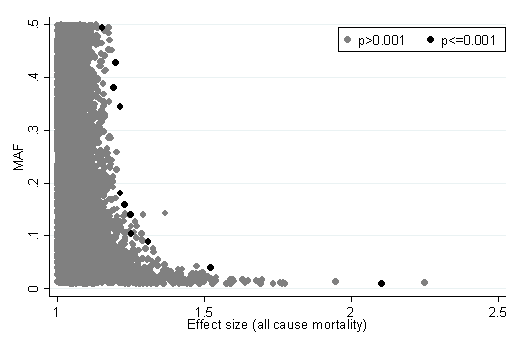 | 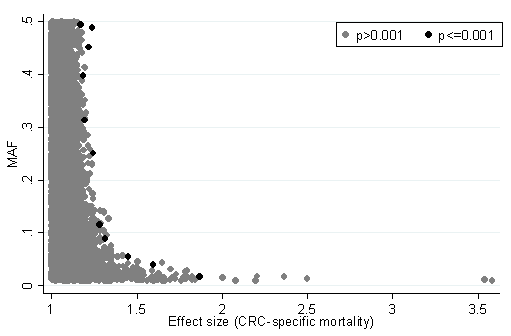 |

**Supplementary Figure 2** Relationship between effect size (for all-cause mortality in plot A and CRC-specific mortality in plot B) and minor allele frequency.


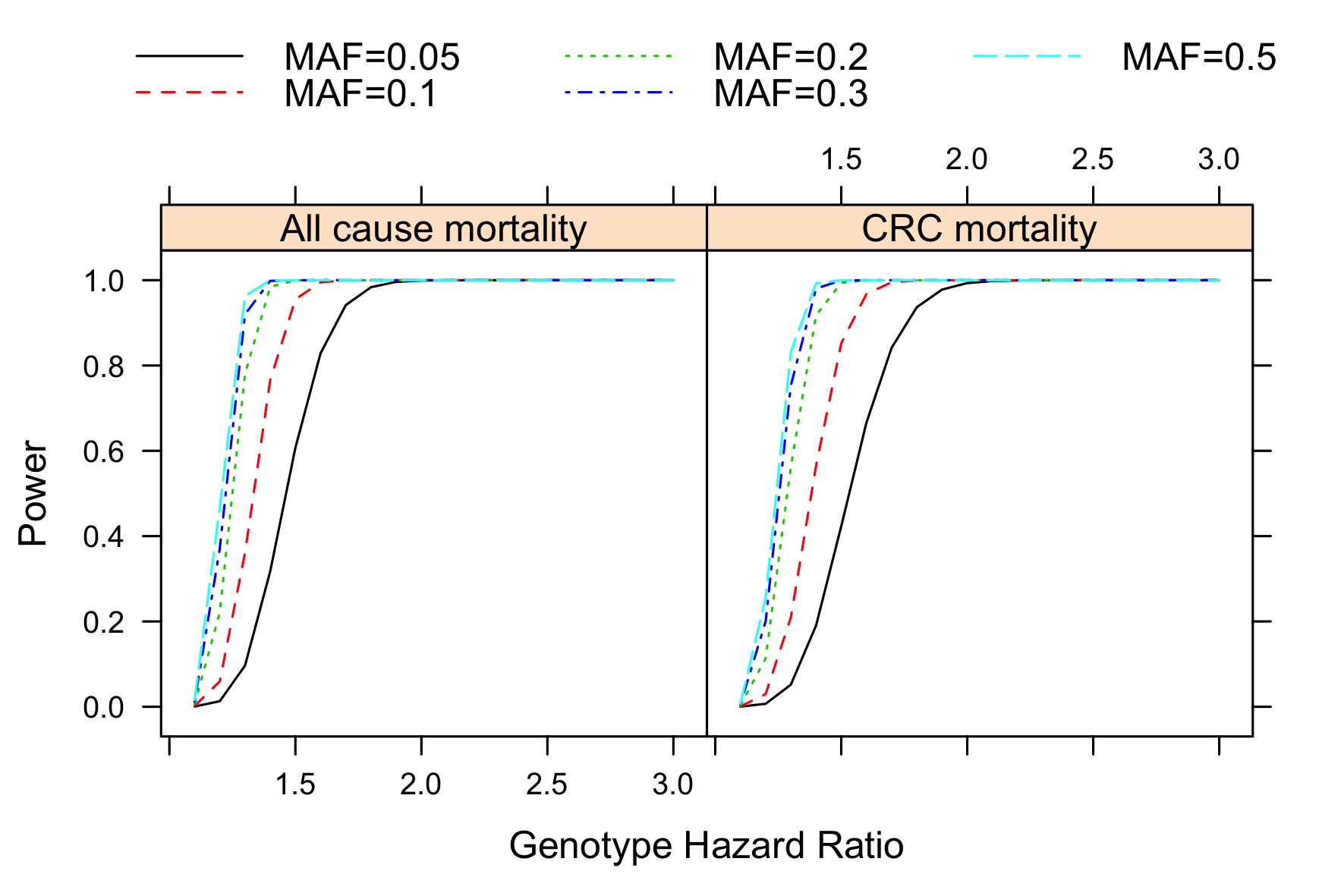


**Supplementary Figure 3** Power to detect nsSNP in all-cause mortality and CRC mortality survival analysis over different expected hazard ratios and for various minor allele frequencies (MAF). We estimated statistical power for a given sample size of combined discovery and replication datasets using the approach described by Owzar et al., 2012 and implemented in “ SurvSNP” statistical package. Power calculation was done for the main effect of genetic variant and log-additive model of inheritance stipulating a P-value of 1e-5 and assuming event rate to be ρ=0.4 (for all-cause mortality) and ρ=0.3 (for CRC mortality).


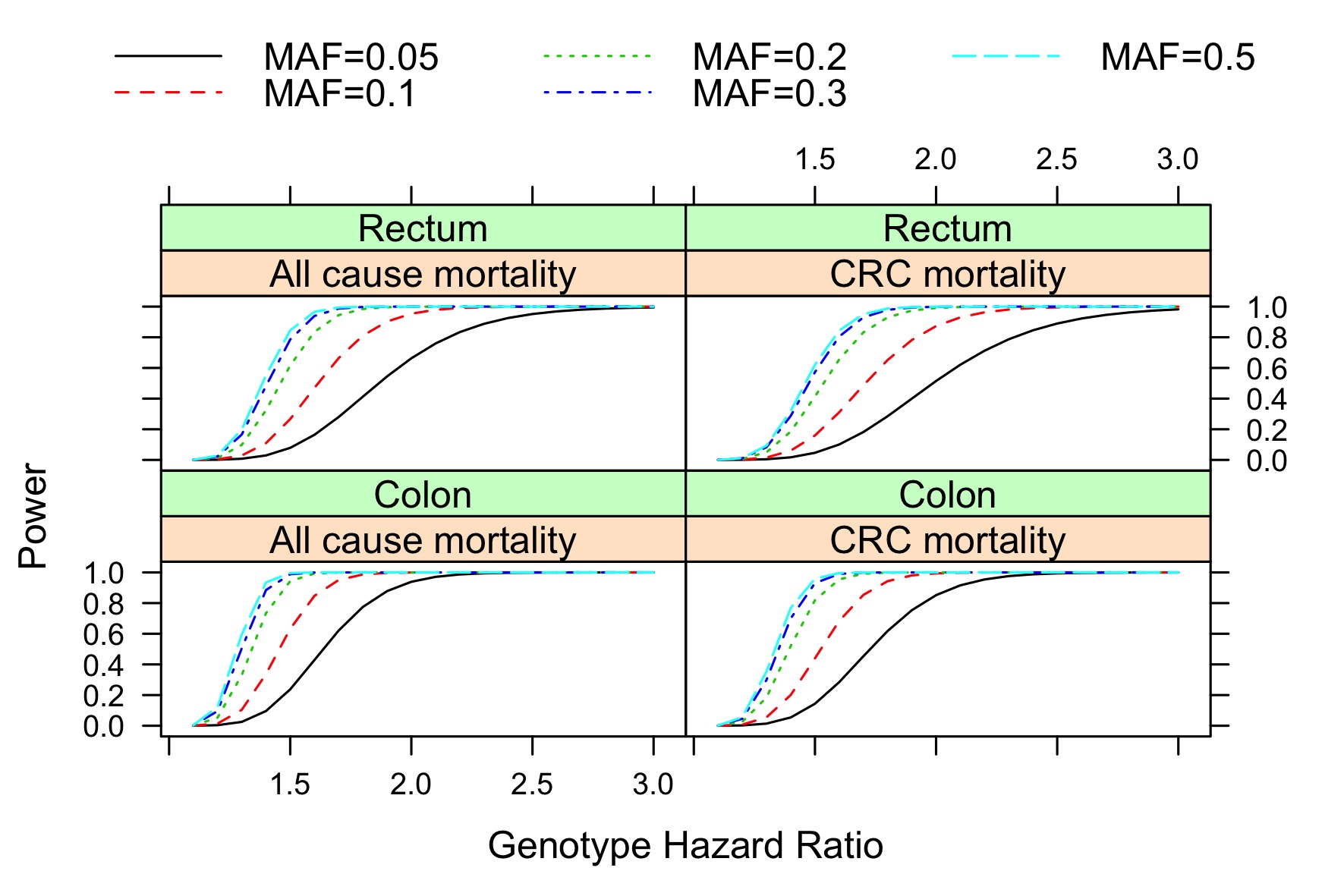


**Supplementary Figure 4** Power to detect nsSNP in all-cause mortality and CRC mortality survival analysis in colon and rectum cancer cases separately over different expected hazard ratios and for various minor allele frequencies (MAF) . We estimated statistical power for a given sample size of combined discovery and replication datasets using approach described by Owzar et al., 2012 and implemented in “ SurvSNP” statistical package. Power calculation was done for the main effect of genetic variant and log-additive model of inheritance stipulating a P-value of 1e-5 and assuming event rate to be ρ=0.4 (for all cause mortality) and ρ=0.3 (for CRC mortality).

1. P-values from the Pearson χ^2^ for categorical variables; from t-test for continuous variables [↑](#footnote-ref-1)
